# Race and sex differences in the association between lifespan glycemic status and midlife cognitive function: the Bogalusa heart study

**DOI:** 10.3389/fpubh.2023.1200415

**Published:** 2023-11-15

**Authors:** Kathryn L. Gwizdala, Lydia A. Bazzano, Robert L. Newton, Owen T. Carmichael

**Affiliations:** ^1^Physical Activity and Ethnic Minority Health Laboratory, Department of Population and Public Health Sciences, Pennington Biomedical Research Center, Louisiana State University System, Baton Rouge, LA, United States; ^2^Tulane Center for Lifespan Epidemiology Research, Tulane University School of Public Health and Tropical Medicine, Tulane University, New Orleans, LA, United States; ^3^Biomedical Imaging Center, Pennington Biomedical Research Center, Louisiana State University System, Baton Rouge, LA, United States

**Keywords:** insulin, glucose, cognitive function, sex, race

## Abstract

**Introduction:**

Glycemic markers throughout life are associated with increased risk of midlife cognitive decline, yet it is unclear whether these associations differ by race and sex.

**Methods:**

This study used cross-sectional analysis of prospectively maintained cohort. 1,295 participants in the Bogalusa Heart Study, a biracial epidemiological cohort located in a micropolitan area core setting, provided fasting plasma insulin (FPI) and glucose (FPG) biannually from 1973 to 2016. Memory, executive function (EF), attention, working memory (WM), and global cognition (GC), collected 2013–2016. Glycemic markers (i.e., FPG, FPI, and HOMA-IR) averaged within lifespan epochs (≤ 20 years, childhood/adolescence (C/A); 21–40 years, early adulthood (EA); and 40–58 years, midlife). Linear regression models were analyzed for each epoch and separate models were analyzed with sex and race, education as a covariate.

**Results:**

Sample was 59% women, 34% African American (AA). Among women, higher C/A FPG was associated with poorer memory and poorer GC. Higher EA FPG was associated with poorer WM. Among men, higher EA HOMA-IR was associated with worse attention. Higher C/A HOMA-IR and FPI were associated with *better* memory, as was higher EA FPI. Among AA, higher C/A FPG was associated with worse attention, EF, and GC. Higher EA HOMA-IR was associated with worse attention. Higher midlife FPI and C/A HOMA-IR were associated with worse WM and EF among White Americans (WAs).

**Discussion:**

Markers indicative of hyperglycemia at different epochs were associated with worse midlife cognition in women, AAs, and WAs; but not in men. Differences in the relationship between lifespan glycemic exposures and midlife cognition could reflect broader health disparities.

## 1 Introduction

Type 2 diabetes is highly prevalent (1), and persons with diabetes (PWDs) have an increased risk of developing dementia and cognitive decline compared to those without the disease (2). An increased risk of dementia and cognitive decline may even extend to individuals exhibiting physiological precursors of diabetes including insulin resistance and hyperglycemia (3, 4). Yet, evidence that treating diabetes lowers the risk of dementia and cognitive decline is highly mixed (5–7), suggesting that our understanding of factors that influence the impact of diabetes-related metabolic processes on risk for dementia and cognitive decline is incomplete.

One such factor is race. Race is associated with differential health exposures that have the potential to influence the association between glycemic control and cognition. African Americans have twice the risk of developing cognitive impairment, and higher rates of diabetes and uncontrolled diabetes, in comparison to their White American counterparts (8). Socioeconomic factors including poorer access to healthcare and environmental stressors such as racial discrimination and neighborhood disadvantage exacerbate health disparities and are hypothesized to contribute to race disparities in cognitive outcomes (9, 10). Biological factors could also contribute to such race disparities: one study found that postmenopausal African American women had less of an increase in cardiorespiratory fitness following exercise training than white women did, for example (11). Older African Americans with diabetes exhibit a greater decline in circulating glucose levels prior to dementia diagnosis than corresponding White Americans with diabetes as well (12). In addition, at least one study has suggested that the relationship between diabetes and cognitive decline is stronger in African Americans compared to White Americans (8). Unfortunately, the evidence base supporting race differences, between African Americans and White Americans, in associations between glycemic control and cognition is small due to a long history of African American under-representation in health and aging research (13). For these reasons, it is imperative to assess if there are differences between African Americans and White Americans in the relationship between glycemic exposures and cognitive outcomes.

Sex is an additional factor that has the potential to influence the association between glycemic control and cognition. Women have higher risk for late life cognitive decline, dementia, AD, and vascular dementia than men (14, 15). However, a recent trial reported that women with diabetes had a *lower* risk for cognitive decline and dementia than corresponding men with diabetes (16), suggesting that sex differences in risk of cognitive outcomes may be modulated by glycemic control. Differences in endogenous sex hormones could also contribute to sex differences in glycemic-cognition relationships, as sex hormones have been found to modulate glycemic control and cognitive outcomes differently in men and women (17, 18). Finally, psychosocial and lifestyle factors that commonly differ by sex, such as education, occupation, or physical activity, may contribute to cognitive decline independently of, or interactively with, glycemic control (19). As a result, examining sex differences in relationships between glycemic exposures and cognitive outcomes is critically important to understand modifiers of the glycemic-cognitive relationship.

Finally, glycemic control-cognition relationships could be influenced by when in the lifespan glycemic control is measured. Most studies are cross-sectional or lack measurements from distal epochs of the lifespan, i.e., studies of middle aged and older adults generally lack measurements from youth and young adulthood. These studies generally suggest that poorer midlife to late life glycemic control is associated with a greater risk of late life cognitive decline (20, 21). A small number of cross sectional studies suggest that obese adolescents with type 2 diabetes have worse cognitive functioning and brain outcomes than corresponding metabolically healthy adolescents do (22, 23). Studies that relate early-life metabolic exposures to midlife cognitive outcomes are scarce, despite initial evidence that such exposures may affect the brain (24). This evidence suggests that relationships between midlife or late-life glycemic control and late-life cognitive function may be modified by glycemic control earlier in life, and therefore motivate further investigation of early life glycemic control and cognition later in life.

This study aimed to evaluate race and sex differences in the association between childhood to midlife glycemic markers and midlife cognitive function in a biracial population-based epidemiological cohort located in a micropolitan area core (25). The cohort was composed of individuals who self-identified as either White or African American. Within the framework of (26), we focused on Question 3: race and sex differences in the relationship between exposure (glycemic markers) and outcome (cognitive function). Together with a rigorous analysis of race and sex differences in the prevalences of poor glycemic control (Question 2) and low cognitive function (Question 1) in this cohort, our analysis could contribute to a better understanding of the possible impact that enhancing glycemic control within specific race and sex groups could theoretically have on disparities in cognitive function. We hypothesized that worse glycemic markers early in life would be associated with worse cognitive function in midlife, but that relationships between worse glycemic markers and worse cognitive function would be more severe among women and African Americans in comparison to men and White Americans.

## 2 Methods

### 2.1 Study design and population

**Figure 1:**
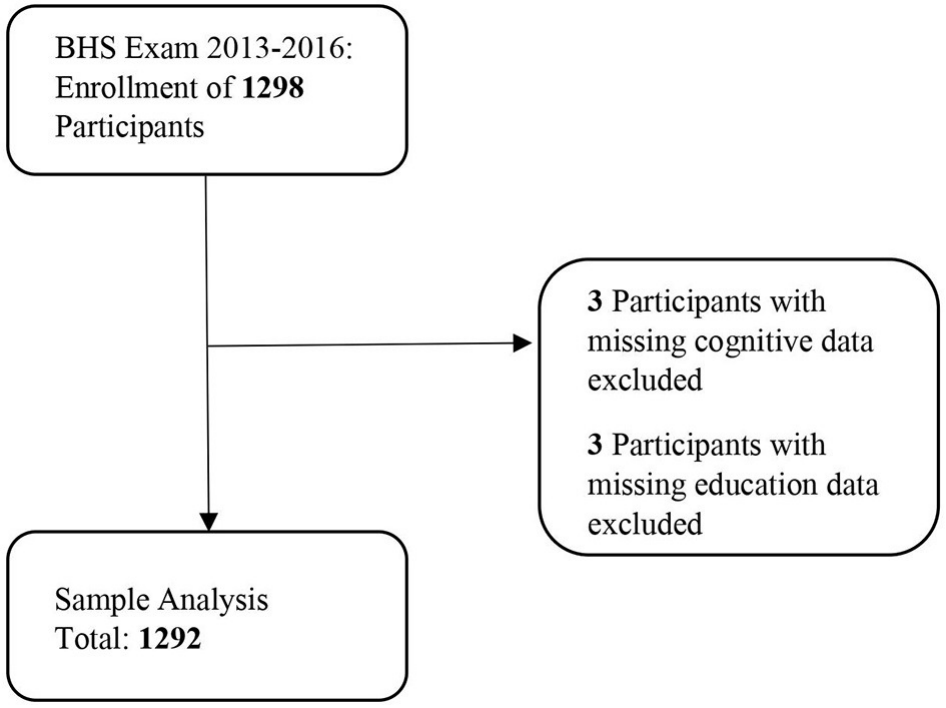
Study flow diagram.

Participants were enrolled in the Bogalusa Heart Study, a biracial population-based cohort of self-identified White and African Americans in Louisiana located in a micropolitan area core (25, 27). Individuals have been followed from childhood to adulthood on roughly a biannual basis for over 40 years. 1298 participants took part in the cognitive test visits between 2013 and 2016. Six individuals were removed from analysis for a total of 1,292 due to missing cognitive tests and education data (Shown in Figure 1). The Six excluded individuals were similar to the full sample (*n* = 1,292) which can be seen in the Supplementary Table 1.

### 2.2 Standard protocol approvals, registrations, and patient consents

This study was approved by the Institutional Review Board at Tulane University and all participants provided informed consent.

### 2.3 Glycemic markers variables

Fasting plasma glucose (FPG) and insulin (FPI) collection, as well as processing procedures, have been described previously (28). Briefly, plasma insulin was analyzed using a radioimmunoassay procedure (Phadebas; Pharmacia Diagnostics, Piscataway, NJ) and plasma glucose was quantified via a Beckman glucose analyzer. Insulin and glucose measurements began during the 4th round of visits in 1976. Insulin resistance was characterized via the homeostasis model assessment of insulin resistance (HOMA-IR) utilizing the following formula: fasting insulin (μU/mL) x fasting glucose (mmol/L)/22.5 (29). Worse glycemic markers were considered to be higher levels of FPG, and FPI and higher HOMA-IR.

### 2.4 Cognition measures

The cognitive tests consisted of memory measures including logical memory I (narrative memory free recall), logical memory II (long term narrative memory free recall), and logical memory II R (long term memory recognition) from the Wechsler Memory Scale III (30); executive function measures including digit span backward and Trail Making B tests (31, 32), attention including Trail Making A test (32), and working memory including digit span forward (31); and a global cognition composite score created by averaging the z-scores of the domain tests (33).

### 2.5 Covariates

Race, sex, and years of education were included in analyses. Race was self-identified and included White Americans and African Americans. Data on ethnicity (Hispanic vs. non-Hispanic White Americans) was not reported historically by Bogalusa Heart Study participants. Sex was self-identified and included men and women. Years of education was self-reported.

### 2.6 Statistical analysis

The groups of interest were men, women, White Americans, and African Americans. The glycemic marker predictors of interest included FPI, FPG, and HOMA-IR averaged within one epoch of the lifespan. The epochs covered birth to 20 years of age (childhood/adolescence), 21- 40 years of age (early adulthood), and 40–58 years of age (midlife). The cognitive outcomes included logical memory I, logical memory II, logical memory II R, digit span forward and backward, Trail Making A and B tests, and global cognition. The z-scores of the individual tests were utilized for analyses.

Separate linear regression models were run, each of which included one sex or race group of interest, had one glycemic marker predictor of interest, had one cognitive outcome of interest, and had years of education as a covariate (with sex as a covariate for models grouped by race and race as a covariate for models grouped by sex). Modeling sex and race groups separately allows for the relationship between glycemic markers and cognitive outcomes to have different estimates (slopes), whereas controlling for these variables pools the estimate of these two groups (i.e., men/women or African American/White American) together and only allows the intercept to be different. R pseudocode for an example model is as follows: lm (formula = Global Cognition ~ Mean Glucose [early adulthood epoch] + Race + Years of Education). We considered including household income as a proxy measure of socioeconomic status into these models, but co-linearity between income and education was high enough to warrant concern about model stability. Models that included income instead of education as a covariate provided similar parameter estimates and goodness of fit compared to those that included education (data not shown). Models that included education are shown in the Results section because that variable was available from a larger sample of individuals. Each model was evaluated with an F test for model fit and a double-sided *p*-value threshold of 0.05.

Outliers among the cognitive and glycemic variables (i.e., outliers were above the 75th or below the 25th percentile by a factor of 3 times the interquartile range) were identified and removed. Outliers were removed from the glycemic variables in each lifespan epoch: childhood/adolescence (31 insulin, 20 glucose, 36 HOMA-IR outliers removed), early adulthood (39 insulin, 41 glucose, 47 HOMA-IR outliers), and midlife (12 insulin, 72 glucose, 15 HOMA-IR outliers). No outliers removed for the global cognition composite score, logical memory I and II, and digit span backwards. There were 4 outliers removed for logical memory II recognition, 1 outlier removed for digit span forward, 12 outliers removed for Trail Making Test A and 16 outliers removed for Trail Making Test B.

Reported model estimates are interpreted as 1 unit of glycemic predictor change corresponding to the reported units of change in the cognitive score (e.g., a reported estimate of −0.5 would mean 1 unit of glycemic predictor change corresponds to −0.5 units of cognitive score change). Additional analyses were run utilizing models containing both childhood/adolescence and early adulthood glycemic marker epochs to clarify whether they each have an independent influence on midlife cognitive function. These analyses followed the same structure as the primary analyses above with the exception that instead of having one time epoch in the model, there were two-time epochs. An example model is as follows, lm (formula = Global Cognition ~ Mean Glucose [20 and under years old Epoch] + Mean Glucose [21 to 40 years old Epoch] + Race + Years of Education). To adjust for multiple comparisons, false discovery rate was computed based on all models that showed initial significance (34). R version 4.0.3 for Windows was used to estimate all statistical models, with stats, jtools, psych, and ggplot packages used to calculate model summaries and for graphing.

### 2.7 Data availability

These data are available upon request to the BHS Steering Committee via the Center for Lifespan Epidemiology. Data request and sharing procedures are available on the website www.clersite.org. Part of these data are archived at the NHLBI BioLINCC data repository and may also be requested there.

## 3 Results

### 3.1 Participants

Characteristics of the sample are summarized in Tables 1, 2 (cognitive outcomes only). The mean age at the time of cognitive testing was 48.2 ± 5.24 years. 59% of the sample was women, and 34 and 66% were African American and White Americans, respectively.

**Table 1:** Participant characteristics: demographic and metabolic.

**Measure**	**Overall**	**Men**	**Women**	***p*-value, 95% CI**	**White American**	**African American**	***p*-value, 95% CI**
Sample size	1292	530	762	-	848	444	-
Sex (% Women)	59%	-	-	-	57%	63%	-
Race (% African American)	34%	31%	37%	-	-	-	-
Education	13.32 ± 2.48	13.09 ± 2.44	13.47 ± 2.5	***p** **<*** **0.01, (−0.65**, **−0.11)**	13.67 ± 2.6	12.64 ± 2.09	***p** **<*** **0.001, (0.77, 1.3)**
**Childhood/Adolescence Epoch (** ≤ **20 years)**							
Glucose (mg/dL)	84.57 ± 7.01 *n =* 1,229	86.66 ± 7.05 *n =* 504	83.11 ± 6.6 *n =* 725	***p** **<*** **0.001, (2.76, 4.33)**	85.09 ± 6.53 *n =* 797	83.6 ± 7.73 *n =* 432	***p** **<*** **0.001, (0.64, 2.36)**
Insulin (mg/dL)	13.31 ± 9.28 *n =* 938	12.9 ± 8.78 *n =* 364	13.57 ± 9.58 *n =* 574	*p >* 0.05, (−1.86, 0.53)	12.49 ± 7.95 *n =* 596	14.73 ± 11.09 *n =* 342	***p** **<*** **0.01**, (–**3.58**, **−0.90)**
HOMA-IR	2.79 ± 2.18 *n =* 938	2.76 ± 1.98 *n =* 364	2.81 ± 2.3 *n =* 574	*p >* 0.05, (−0.33, 0.23)	2.63 ± 1.88 *n =* 596	3.07 ± 2.61 *n =* 342	***p** **<*** **0.01, (−0.75**, **−0.12)**
**Early adulthood Epoch (21–40 years)**							
Glucose (mg/dL)	85.87 ± 18.06 *n =* 1,108	89.03 ± 19.09 *n =* 437	83.81 ± 17.07 *n =* 671	***p** **<*** **0.001, (3.01, 7.43)**	84.24 ± 13.06 *n =* 733	89.05 ± 24.83 *n =* 375	***p** **<*** **0.001**, (–**7.50**, **−2.12)**
Insulin (mg/dL)	12.44 ± 9.7 *n =* 1,071	11.9 ± 9.23 *n =* 417	12.79 ± 9.98 *n =* 654	*p >* 0.05, (−2.06, 0.28)	11.18 ± 6.44 *n =* 722	15.05 ±13.9 *n =* 349	***p** **<*** **0.001**, (–**5.41**, **−2.34)**
HOMA-IR	2.77 ± 3.07 *n =* 1,057	2.78 ± 3.43 *n =* 407	2.76 ± 2.83 *n =* 650	*p >* 0.05, (−0.38, 0.42)	2.39 ± 1.82 *n =* 710	3.55 ± 4.6 *n =* 347	***p** **<*** **0.001, (−1.67**, **−0.66)**
**Midlife (**>**40 years)**							
Glucose (mg/dL)	103.37 ± 34.89 *n =* 1,173	105.35 ± 31.9 *n =* 477	102.01 ± 36.75 *n =* 696	*p >* 0.05, (−0.62, 7.30)	102.44 ± 31.58 *n =* 785	105.24 ± 40.75 *n =* 388	*p >* 0.05, (−7.43, 1.83)
Insulin (mg/dL)	12.93 ± 9.99 *n =* 472	13.15 ± 9.74 *n =* 187	12.78 ± 10.17 *n =* 285	*p >* 0.05, (−1.47, 2.20)	12.31 ± 9.67 *n =* 324	14.29 ±10.58 *n =* 148	*p =* 0.05, (−4, 0.03)
HOMA-IR	3.21 ± 3 *n =* 470	3.27 ± 2.77 *n =* 187	3.17 ± 3.15 *n =* 283	*p >* 0.05, (−0.44, 0.64)	2.97 ± 2.79 *n =* 324	3.74 ± 3.37 *n =* 146	***p** **<*** **0.05, (−1.40**, **−0.15)**

For continuous measures, mean ± standard deviation is shown, as are the *p* values for *t* tests comparing men and women; and *t* tests comparing White Americans and African Americans. 95% confidence intervals for the men mean value minus women mean value, and White American mean value minus African American mean value, are also shown. Bold values indicate *p*-values and 95% CIs of the statistically significant *t*-tests conducted.

**Table 2:** Participant characteristics: cognitive outcomes.

**Measure**	**Overall**	**Men**	**Women**	***p*-value, 95% CI**	**White American**	**African American**	***p*-value, 95% CI**
Global cognitive function	0.01 ± 5.36	−0.75 ± 5.12	0.53 +/- 5.47	***p** **<*** **0.001, (−1.87**, **−0.70)**	1.27 ± 4.97	−2.41 ± 5.26	***p** **<*** **0.001, (3.08, 4.28)**
Logical memory I	0 ± 1	−0.08 ± 1	0.06 +/- 0.99	***p** **<*** **0.05, (−0.25**, **−0.03)**	0.18 ± 0.99	−0.34 ± 0.93	***p** **<*** **0.001, (0.41, 0.63)**
Logical memory II	0 ±1	−0.11 ± 1	0.08 +/- 0.99	***p** **<*** **0.001, (−0.31**, **−0.08)**	0.19 ± 0.99	−0.36 ± 0.91	***p** **<*** **0.001, (0.44, 0.66)**
Logical memory II r	0 ± 0.99	−0.11 ± 1.05	0.09 +/- 0.95	***p** **<*** **0.001, (−0.31**, **−0.09)**	0.17 ± 0.92	−0.31 ± 1.06	***p** **<*** **0.001, (0.36, 0.60)**
Digit span forward	0 ± 1	0.02 ± 0.99	−0.01 +/- 1.01	*p >* 0.05, (−0.08, 0.14)	0.12 ± 0.96	−0.24 ± 1.03	***p** **<*** **0.001, (0.25, 0.48)**
Digit span backward	0 ± 1	−0.04 ±1.02	0.03 ± 0.99	*p >* 0.05, (−0.19, 0.036)	0.2 ± 1	−0.38 ± 0.88	***p** **<*** **0.001, (0.48, 0.70)**
Trail making A	0 ± 1	0.08 ± 0.93	−0.06 ± 1.03	***p** **<*** **0.05, (0.023, 0.24)**	−0.12 ± 0.82	0.22 ± 1.24	***p** **<*** **0.001, (−0.47**, **−0.21)**
Trail making B	0 ± 1	0.07 ± 0.96	−0.05 ± 1.02	***p** **<*** **0.05, (0.005, 0.23)**	−0.17 ± 0.87	0.32 ± 1.14	***p** **<*** **0.001, (−0.61**, **−0.40)**

For continuous measures, mean ± standard deviation is shown, as are the *p* values for *t* tests comparing men and women; and *t* tests comparing White Americans and African Americans. 95% confidence intervals for the men mean value minus women mean value, and White American mean value minus African American mean value, are also shown. Bold values indicate *p*-values and 95% CIs of the statistically significant *t*-tests conducted.

### 3.2 Sex stratified analyses

**Figure 2:**
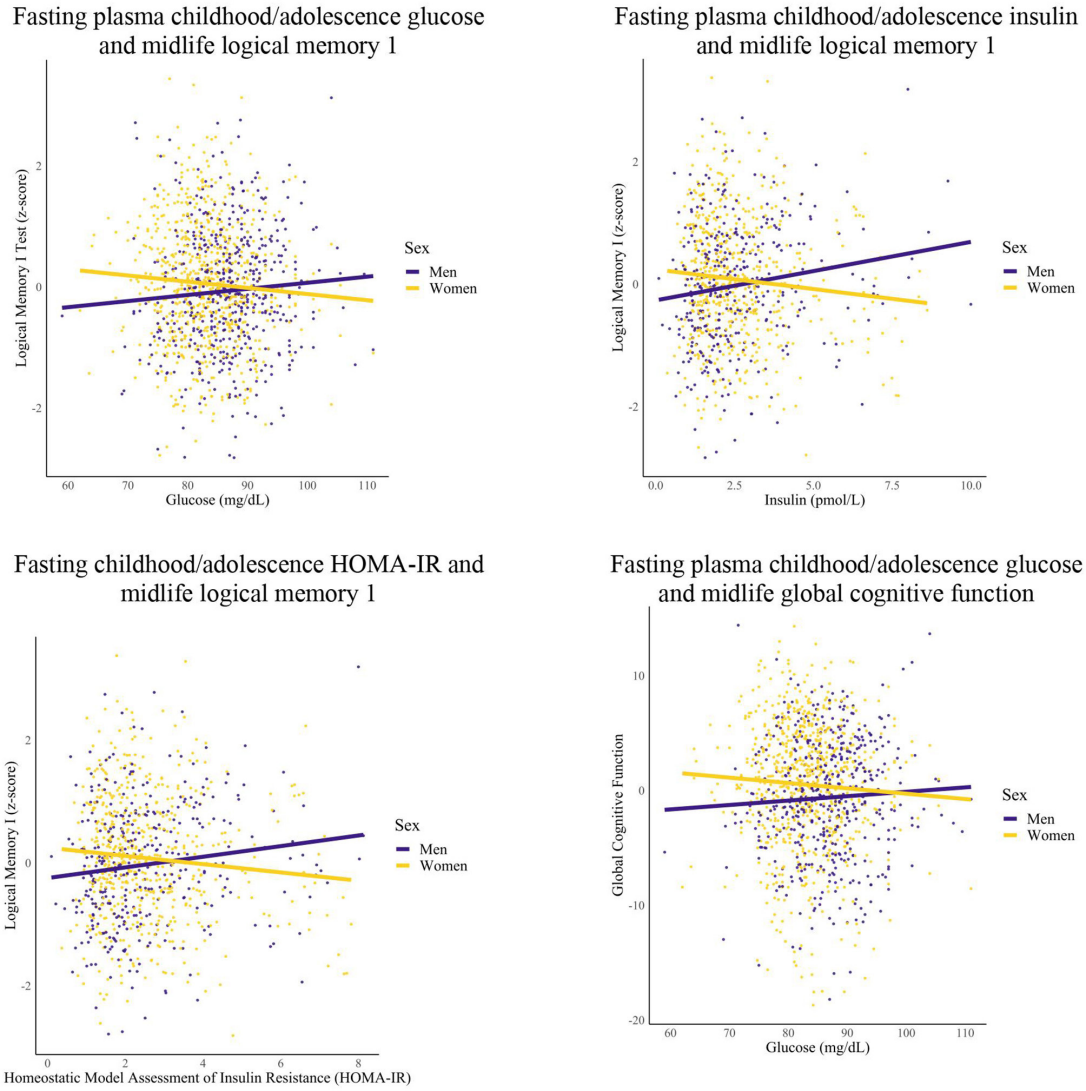
Differences in childhood/adolescence glycemic markers-midlife cognition relationships by sex. Linear regression models were conducted for each epoch. Covariates included years of education and race.

Model estimates and *p*-values for statistically significant effects in the sex-specific models are reported in Table 3. Qualitative descriptions of those results are described here. Selected associations from these analyses are represented visually in Figure 2. Note that Trails A and B have been transformed so that higher scores indicate better performance.

**Table 3:** Sex stratified analyses: glycemic marker cognition estimates.

**Group of interest**	**Model components** ^**a**^			**Results**	
**Sex**	**Glycemic time epoch** ^b^	**Glycemic predictor**	**Cognitive outcome task**	**Model estimate**	***p*** **-value**
Women	Childhood/adolescence	FPG^c^	Logical memory 1	−0.01	0.024
	Childhood/adolescence	FPG	Logical memory 2 recognition	−0.01	0.0413
	Childhood/adolescence	FPG	Global cognition	−0.07	0.024
	Early adulthood	FPG	Digit span forward	−0.01	0.0378
Men	Childhood/adolescence	HOMA-IR	Logical memory 1	0.09	0.024
	Childhood/adolescence	FPI^d^	Logical memory 1	0.02	0.024
	Childhood/adolescence	HOMA-IR	Logical memory 2	0.08	0.0378
	Childhood/adolescence	FPI	Logical memory 2	0.02	0.0366
	Early adulthood	FPI	Logical memory 2	0.02	0.0416
	Early adulthood	HOMA-IR	Trail making test A	−0.08	0.024

^a^Linear regression models were conducted for each epoch. Covariates included years of education, sex for models grouped by race and race for models grouped by sex. ^b^Glycemic time epoch indicates what time point in the lifespan the glycemic predictor was collected in for that reported model estimate. ^c^FPG, Fasting plasma glucose. ^d^FPI, Fasting plasma insulin.

Among women, greater FPG within the childhood/adolescence epoch was associated with worse memory (1 unit increase FPG, 0.01 unit decrease logical memory 1 and logical memory 2 recognition) and global cognition performance (1 unit increase FPG, 0.07 unit decrease global cognition) in midlife. Additionally, women had greater FPG within the early adulthood epoch that was associated with worse midlife Digit Span Forward performance (1 unit increase FPG, 0.01 unit decrease digit span forward performance).

Among men, greater HOMA-IR and FPI levels within the childhood/adolescence epochs were associated with greater memory performance (1 unit increase HOMA-IR, 0.09 unit increase logical memory 1 and 0.08 unit increase logical memory 2; 1 unit increase FPI, 0.02 unit increase logical memory 1 and 2 performance). Furthermore, men had greater early adulthood FPI that was associated with greater midlife memory performance (1 unit increase FPI, 0.02 unit increase logical memory 2), but higher HOMA-IR in early adulthood was associated with worse Trail Making Test A performance (1 unit increase HOMA-IR, 0.08 unit decrease Trail Making Test A performance). After additional analyses, the significant association between early adulthood FPI and midlife memory performance was attenuated for men.

For women, no significant relationships were found for the childhood/adolescence and early adulthood epochs for FPI and HOMA-IR (data not shown) with select significant relationships for FPG (as reported in this section). For men, no significant relationships were found for the childhood/adolescence and early adulthood epochs for FPG (data not shown) with select significant relationships found for FPI and HOMA-IR (as reported in this section). For both groups, no significant relationships were found for any of the metabolic markers within the midlife epoch.

### 3.3 Race stratified analyses

**Figure 3:**
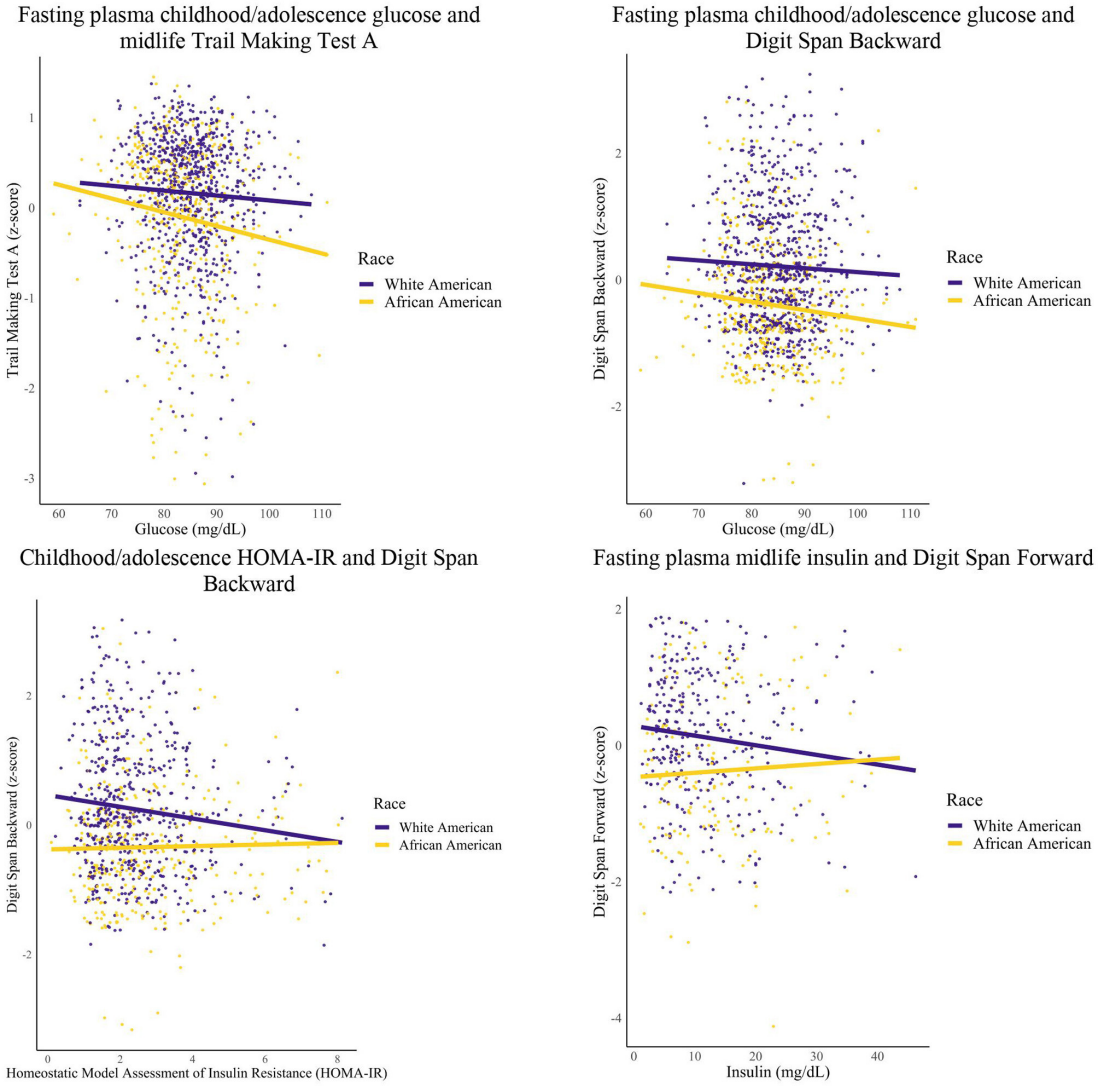
Glycemic markers and midlife cognition: race groups. Linear regression models were conducted for each epoch. Covariates included years of education and sex.

Model estimates and *p*-values for statistically significant effects in the race-specific models are reported in Table 4. Qualitative descriptions of those results are described here. Selected associations from these analyses are represented visually in Figure 3. As above, Trails A and B have been transformed so that higher scores indicate better performance.

**Table 4:** Race stratified analyses: glycemic marker cognition estimates.

**Group of Interest**	**Model components** ^**a**^			**Results**	
**Race**	**Glycemic time epoch** ^b^	**Glycemic predictor**	**Cognitive outcome task**	**Model estimate**	***p*** **-value**
African Americans	Childhood/adolescence	FPG^c^	Digit span backward	−0.01	0.0416
	Childhood/adolescence	FPG	Trail making test A	−0.01	0.0378
	Childhood/adolescence	FPG	Global cognition	−0.06	0.049
	Early adulthood	HOMA-IR	Trail making test A	−0.07	0.0378
White Americans	Midlife	FPI^d^	Digit span forward	−0.01	0.0416
	Childhood/adolescence	HOMA-IR	Digit span backward	−0.08	0.024

^a^Linear regression models were conducted for each epoch. Covariates included years of education, sex for models grouped by race and race for models grouped by sex. ^b^Glycemic time epoch indicates what time point in the lifespan the glycemic predictor was collected in for that reported model estimate. ^c^FPG, Fasting plasma glucose. ^d^FPI, Fasting plasma insulin.

Among African Americans, greater FPG within the childhood/adolescent epoch was associated with worse Digit Span Backward (1 unit increase FPG, 0.01 unit decrease Digit Span Backward performance), Trail Making Test A (1 unit increase FPG, 0.01 unit decrease Trail Making A performance), and global cognition scores (1 unit increase FPG, 0.06 unit decrease global cognition). Additionally, African Americans had greater HOMA-IR within the early adulthood epoch that was associated with worse Trail Making Test A scores (1 unit increase HOMA-IR, 0.07 unit decrease Trail Making A performance).

Among White Americans, greater levels of FPI during midlife was associated with worse Digit Span Forward scores (1 unit increase of FPI, 0.01 unit decrease Digit Span Forward performance). Furthermore, White Americans had greater HOMA-IR within the childhood/adolescent epoch that was associated with worse Digit Span Backward scores (1 unit increase HOMA-IR, 0.08 unit decrease Digit Span Backward performance).

For White Americans, no significant relationships were found for the childhood/adolescence epoch for FPG (data not shown) with one significant relationship found for HOMA-IR (as reported in this section), and for the midlife epoch, no significant relationships were found for HOMA-IR and FPG (data not shown) with one significant relationship found for FPI (as reported in this section). Additionally for White Americans, there were no significant relationships found for any metabolic marker for the early adulthood epoch. For African Americans, no significant relationships were found for the childhood/adolescence epoch for HOMA-IR (data not shown) with select significant relationships found for FPG (as reported in this section), and for the early adulthood epoch, no significant relationships were found for FPI and FPG (data not shown) with one significant relationship found for HOMA-IR (as reported in this section). Additionally for African Americans, no significant relationships were found for any of the metabolic markers within the midlife epoch. For both groups, no significant relationships were found for the childhood/adolescence epoch for FPI.

## 4 Discussion

In support of our hypotheses, poorer glycemic markers in childhood/adolescence and early adulthood were associated with worse midlife cognitive function in certain groups (i.e., women, African Americans, and White Americans). In addition, poorer midlife fasting plasma insulin was associated with worse working memory among White Americans. However, contradicting our hypotheses, poorer glycemic markers in childhood/adolescence and early adulthood were associated with *better* cognitive functioning among men. Overall, the pattern of findings suggests that cognitive outcomes associated with glycemic dysfunction differ significantly by sex and that childhood/adolescence is a key time period for glycemic markers.

The finding that better glycemic markers are associated with better cognitive function in certain groups is consistent with several prior observational and interventional studies (20, 21, 35). The finding is also consistent with a prior finding from this cohort that better glycemic markers are associated with better brain outcomes (24). Conversely, the opposite association observed in other subgroups in this study—better glycemic markers being associated with *worse* cognition—has also been supported in prior studies (36, 37). In addition, this same relationship between glycemic markers and cognitive function within certain subgroups has been reported to be null in some studies (35) and U-shaped in others (38). Together, these findings suggest that the relationship between glycemic markers and cognitive functioning is complex with more longitudinal investigations exploring the nuances of different subgroups needed to fully understand the nature of it.

Sex differences in the relationship between glycemic markers and cognitive function could be driven by sex differences in behavioral and biological contributors to glycemic markers and cognitive function. Women exhibit more physical inactivity than men in multiple parts of the lifespan, and men have traditionally exhibited higher levels of physical activity due to greater engagement in physically demanding jobs (39, 40). This greater level of physical activity attainment may have buffered the brains of the male participants against the negative effects of poorer glycemic markers (41). Alternatively, differing temporal patterns of endogenous sex hormone levels between sexes, including during puberty (42), could modify these associations, as sex hormone levels are believed to affect glycemic markers and diabetes risk (17) as well as possibly dementia risk (43). This difference in timing could fail to safeguard the brains of women, in comparison to men, from the effects of glycemic markers on cognition. Future work is needed to better understand the biological basis of observed sex differences in relationships between lifespan glycemic markers and cognition.

We did not find any prominent differences between African Americans and White Americans for the glycemic marker cognition relationships as the corresponding effects were similar and going in the same direction despite significant vs. non-significant relationships varying between races. This contrasts with our hypothesis that cognitive effects of poorer glycemic markers would be especially prominent among African Americans due to differences in biological factors (e.g., APOE genotype), intervention responses (e.g., poorer aerobic outcomes) and environmental stressors (e.g., structural racism, poorer access to healthcare, poorer air quality, etc.) in comparison to White Americans, each of which could confer a greater risk of poor cognitive outcomes (9, 11, 44, 45). The finding also contrasts with prior data that addressed a similar question (8). Within the current small literature, there is one study that also observed no differences in glycemic-cognition relationships between race groups (46). Our lack of such race differences may be due in part to our inclusion of education as a covariate in statistical models, as education may have served as a proxy for some of the aforementioned environmental stressors. Indeed, prior studies have suggested that controlling for education can attenuate race differences in cognition poverty level (47, 48). Due to the nuances of the glycemic-cognition relationship, more longitudinal investigations of race differences in matched samples of AA and WA are warranted.

We found that glycemic markers in earlier epochs of the lifespan was associated with cognitive function in midlife. This aligns with previous findings in this same cohort showing that higher FPG in early life was associated with poorer brain health in midlife (24). The finding also aligns with previous studies of youth with Type 1 Diabetes (T1D) who went on to have worse cognitive function as young adults (an average of 5.5 years and 12 years later respectively) than youth without T1D did (49, 50). The results could be driven by the sensitivity of the brain during childhood and adolescence years to stressors such as impaired glucose markers (51). Future studies should utilize cognitive measures at multiple times points across the lifespan to provide a more complete picture of the dynamic effects that fluctuations in glycemic markers have on cognitive function throughout the lifespan. Furthermore, this study's findings also suggest that early life is a key time for future interventions on glycemic markers.

This study has notable strengths. The participants provided glycemic data covering approximately 40 years of the lifespan, thus enabling novel assessment of epoch-dependent relationships with cognition. The racial and socioeconomic diversity of the cohort is an additional strength, although generalization of results outside of this micropolitan area core setting should be approached with caution. One weakness is that glycemic marker measures did not include modern measures such as Hemoglobin A1c (Hba1c), which had not been developed until after the initiation of the cohort.

## 5 Conclusions

The findings from the current study are one of the first to suggest that early life glycemic markers are important determinants of future cognitive outcomes in this semi-rural, biracial cohort. Other important findings indicate that the relationship between glycemic markers and cognitive outcomes was similar across racial groups and in the expected direction, however these differed by sex group. Our results point to future directions which should include measures of brain function (e.g., functional MRI), glucose metabolism (e.g., fluorodeoxyglucose positron emission tomography or FDG-PET), and neurodegenerative disease (e.g., amyloid PET) to clarify mechanisms underlying sex and race differences in glycemic markers effects on the brain across the lifespan. Improving this understanding could provide insight into how to individually tailor glycemic marker interventions so that they lessen adverse brain consequences.

## Data availability statement

The raw data supporting the conclusions of this article will be made available by the authors, without undue reservation.

## Ethics statement

The studies involving human participants were reviewed and approved by the Institutional Review Board of Tulane University. The patients/participants provided their written informed consent to participate in this study.

## Author contributions

KG drafting and revision of the manuscript for content, including medical writing for content, study concept or design, and analysis or interpretation of data. LB drafting and revision of the manuscript for content, including medical writing for content, and major role in the acquisition of data. RN and OC drafting and revision of the manuscript for content, including medical writing for content, and analysis or interpretation of data. All authors contributed to the article and approved the submitted version.

## References

[1] Andes LJ Cheng YJ Rolka DB Gregg EW Imperatore G. Prevalence of prediabetes among adolescents and young adults in the United States, 2005-2016. JAMA Pediatr. (2019) 174:e194498. 10.1001/jamapediatrics.2019.4498 31790544 PMC6902249

[2] Rawlings AM Sharrett AR Albert MS Coresh J Windham BG Power MC. The association of late-life diabetes status and hyperglycemia with incident mild cognitive impairment and dementia: The ARIC study. Diabetes Care. (2019) 42:1248–54. 10.2337/dc19-0120 31221696 PMC6609963

[3] Bruehl H Sweat V Hassenstab J Polyakov V Convit A. Cognitive impairment in nondiabetic middle-aged and older adults is associated with insulin resistance. J Clin Exp Neuropsychol. (2010) 32:487–93. 10.1080/13803390903224928 20524222 PMC3116728

[4] Tan ZS Beiser AS Fox CS Au R Himali JJ Debette S. Association of metabolic dysregulation with volumetric brain magnetic resonance imaging and cognitive markers of subclinical brain aging in middle-aged adults: the Framingham Offspring study. Diabetes Care. (2011) 34:1766–70. 10.2337/dc11-0308 21680719 PMC3142014

[5] Campbell JM Stephenson MD de Courten B Chapman I Bellman SM Aromataris E. Metformin use associated with reduced risk of dementia in patients with diabetes: a systematic review and meta-analysis. J Alzheimers Dis. (2018) 65:1225–36. 10.3233/JAD-180263 30149446 PMC6218120

[6] McMillan JM Mele BS Hogan DB Leung AA. Impact of pharmacological treatment of diabetes mellitus on dementia risk: systematic review and meta-analysis. BMJ Open Diabetes Res Care. (2018) 6:e000563. 10.1136/bmjdrc-2018-000563 30487973 PMC6254737

[7] Sastre AA Vernooij RW Harmand MGC Martínez G. Effect of the treatment of Type 2 diabetes mellitus on the development of cognitive impairment and dementia. Cochrane Database Syst Rev. (2017) 6:1–5. 10.1002/14651858.CD003804.pub2 PMC6481422 28617932

[8] Mayeda ER Haan MN Neuhaus J Yaffe K Knopman DS Sharrett AR. Type 2 diabetes and cognitive decline over 14 years in middle-aged African Americans and whites: the ARIC Brain MRI Study. Neuroepidemiology. (2014) 43:220–7. 10.1159/000366506 25402639 PMC4370220

[9] Gee Gilbert C Payne-Sturges Devon C. Environmental health disparities: a framework integrating psychosocial and environmental concepts. Environ Health Perspect. (2004) 112:1645–53. 10.1289/ehp.7074 15579407 PMC1253653

[10] Majoka MA Schimming C. Effect of social determinants of health on cognition and risk of alzheimer disease and related dementias. Clin Ther. (2021) 43:922–9. 10.1016/j.clinthera.2021.05.005 34103175

[11] Swift DL Johannsen NM Lavie CJ Earnest CP Johnson WD Blair SN. Racial differences in the response of cardiorespiratory fitness to aerobic exercise training in Caucasian and African American postmenopausal women. J Appl Physiol. (1985) 114:1375–82. 10.1152/japplphysiol.01077.2012 23471944 PMC3656426

[12] Hendrie HC Zheng M Lane KA Ambuehl R Purnell C Li S. Changes of glucose levels precede dementia in African Americans with diabetes but not in Caucasians. Alzheimers Dement J Alzheimers Assoc. (2018) 14:1572–9. 10.1016/j.jalz.2018.03.008 29678640 PMC6192866

[13] Luebbert R Perez A. Barriers to clinical research participation among African Americans. J Transcult Nurs. (2016) 27:456–63. 10.1177/1043659615575578 25754929

[14] Chatterjee S Peters SAE Woodward M Mejia Arango S Batty GD Beckett N. Type 2 diabetes as a risk factor for dementia in women compared with men: a pooled analysis of 2. 3 million people comprising more than 100,000 cases of dementia. Diabetes Care. (2016) 39:300–7. 10.2337/dc15-1588 26681727 PMC4722942

[15] Rocca WA Mielke MM Vemuri P Miller VM. Sex and gender differences in the causes of dementia: a narrative review. Maturitas. (2014) 79:196–201. 10.1016/j.maturitas.2014.05.008 24954700 PMC4169309

[16] Gong J Harris K Hackett M Peters SA Brodaty H Cooper M. Sex differences in risk factors for cognitive decline and dementia, including death as a competing risk, in individuals with diabetes: results from the ADVANCE trial. Diabetes Obes Metab. (2021) 23:1775–85. 10.1111/dom.14391 33783955

[17] Ding EL Song Y Malik VS Liu S. Sex differences of endogenous sex hormones and risk of type 2 diabetes: a systematic review and meta-analysis. JAMA. (2006) 295:1288. 10.1001/jama.295.11.1288 16537739

[18] Sumien N Cunningham JT Davis DL Engelland R Fadeyibi O Farmer GE. Neurodegenerative disease: roles for sex, hormones, and oxidative stress. Endocrinology. (2021) 162:11. 10.1210/endocr/bqab185 34467976 PMC8462383

[19] Levine DA Gross AL Briceño EM Tilton N Giordani BJ Sussman JB. Sex differences in cognitive decline among US adults. JAMA Netw Open. (2021) 4:e210169–e210169. 10.1001/jamanetworkopen.2021.0169 33630089 PMC7907956

[20] Ravona-Springer R Moshier E Schmeidler J Godbold J Akrivos J Rapp M. Changes in glycemic control are associated with changes in cognition in non-diabetic elderly. J Alzheimers Dis. (2012) 30:299–309. 10.3233/JAD-2012-120106 22426020 PMC3586408

[21] Rawlings AM Sharrett AR Schneider ALC Coresh J Albert M Couper D. Diabetes in midlife and cognitive change over 20 years: the atherosclerosis risk in communities neurocognitive study. Ann Intern Med. (2014) 161:785–93. 10.7326/M14-0737 25437406 PMC4432464

[22] Brady CC Vannest JJ Dolan LM Kadis DS Lee GR Holland SK. Obese adolescents with type 2 diabetes perform worse than controls on cognitive and behavioral assessments. Pediatr Diabetes. (2017) 18:297–303. 10.1111/pedi.12383 27028236

[23] Yau PL Javier DC Ryan CM Tsui WH Ardekani BA Ten S. Preliminary evidence for brain complications in obese adolescents with type 2 diabetes mellitus. Diabetologia. (2010) 53:2298–306. 10.1007/s00125-010-1857-y 20668831 PMC3116653

[24] Carmichael OT Stuchlik P Pillai S Biessels GJ Dhullipudi R Madden-Rusnak A. High-normal adolescent fasting plasma glucose is associated with poorer midlife brain health: bogalusa heart study. J Clin Endocrinol Metab. (2019) 104:4492–500. 10.1210/jc.2018-02750 31058974 PMC6736207

[25] U.S. Department of Agriculture Economic Research Service. Rural-Urban Commuting Area Codes. (2023). Available online at: https://www.ers.usda.gov/data-products/rural-urban-commuting-area-codes/ (accessed June 19, 2023).

[26] Ward JB Gartner DR Keyes KM Fliss MD McClure ES Robinson WR. How do we assess a racial disparity in health? Distribution, interaction, and interpretation in epidemiological studies. Ann Epidemiol. (2019) 29:1–7. 10.1016/j.annepidem.2018.09.007 30342887 PMC6628690

[27] Berenson GS. Bogalusa heart study: a long-term community study of a rural biracial (black/white) population. Am J Med Sci. (2001) 322:267–74. 10.1097/00000441-200111000-00007 11721800

[28] Burke GL Webber LS Srinivasan SR Radhakrishnamurthy B Freedman DS Berenson GS. Fasting plasma glucose and insulin levels and their relationship to cardiovascular risk factors in children: Bogalusa heart study. Metabolism. (1986) 35:441–6. 10.1016/0026-0495(86)90135-6 3517558

[29] Matthews DR Hosker JP Rudenski AS Naylor BA Treacher DF Turner RC. Homeostasis model assessment: insulin resistance and β-cell function from fasting plasma glucose and insulin concentrations in man. Diabetologia. (1985) 28:412–9. 10.1007/BF00280883 3899825

[30] Uttl B Graf P. The Wechsler memory scale-III: validity and reliability. Arch Clin Neuropsychol. (1999) 8:705–6. 10.1093/arclin/14.8.705a

[31] Wechsler D. Wechsler Adult Intelligence Scale, 4th Edn. Worcester, MA: American Psychological Association (2008).

[32] Reitan RM. Trail Making Test: Manual for Administration, Scoring and Interpretation. Bloomington, IN: Indiana University Bloomington. (1956), p. 134.

[33] Harville EW Guralnik J Romero M Bazzano LA. Reproductive history and cognitive aging: the Bogalusa heart study. Am J Geriatr Psychiatry Off J Am Assoc Geriatr Psychiatry. (2020) 28:217–25. 10.1016/j.jagp.2019.07.002 31350162 PMC6942641

[34] Benjamini Y Hochberg Y. Controlling the false discovery rate: a practical and powerful approach to multiple testing. J R Stat Soc Ser B Methodol. (1995) 57:289–300. 10.1111/j.2517-6161.1995.tb02031.x

[35] Carmichael OT Neiberg RH Dutton GR Hayden KM Horton E Pi-Sunyer FX. Long-term change in physiological markers and cognitive performance in type 2 diabetes: the look AHEAD study. J Clin Endocrinol Metab. (2020) 105:12. 10.1210/clinem/dgaa591 32845968 PMC7566388

[36] Bruce DG Davis WA Starkstein SE Davis TM. Mid-life predictors of cognitive impairment and dementia in type 2 diabetes mellitus: the Fremantle diabetes study. J Alzheimers Dis. (2014) 42:S63–70. 10.3233/JAD-132654 24840567

[37] van den Berg E de Craen AJM Biessels GJ Gussekloo J Westendorp RGJ. The impact of diabetes mellitus on cognitive decline in the oldest of the old: a prospective population-based study. Diabetologia. (2006) 49:2015–23. 10.1007/s00125-006-0333-1 16804671

[38] Shorr RI de Rekeneire N Resnick HE Yaffe K Somes GW Kanaya AM. Glycemia and cognitive function in older adults using glucose-lowering drugs. J Nutr Health Aging. (2006) 10:297–301. 16886100

[39] García-Fernández J González-López JR Vilches-Arenas Á Lomas-Campos M de las M. Determinants of physical activity performed by young adults. Int J Environ Res Public Health. (2019) 16:4061. 10.3390/ijerph16214061 31652693 PMC6861903

[40] Guthold R Stevens GA Riley LM Bull FC. Global trends in insufficient physical activity among adolescents: a pooled analysis of 298 population-based surveys with 1·6 million participants. Lancet Child Adolesc Health. (2020) 4:23–35. 10.1016/S2352-4642(19)30323-2 31761562 PMC6919336

[41] Colberg SR Somma CT Sechrist SR. Physical activity participation may offset some of the negative impact of diabetes on cognitive function. J Am Med Dir Assoc. (2008) 9:434–8. 10.1016/j.jamda.2008.03.014 18585646

[42] Marshall WA. Sex differences at puberty. J Biosoc Sci. (1970) 2:31–41. 10.1017/S0021932000023439 5276624

[43] Ford AH Yeap BB Flicker L Hankey GJ Chubb SAP Golledge J. Sex hormones and incident dementia in older men: the health in men study. Psychoneuroendocrinology. (2018) 98:139–47. 10.1016/j.psyneuen.2018.08.013 30144781

[44] Yearby R. Racial disparities in health status and access to healthcare: the continuation of inequality in the United States due to structural racism. Am J Econ Sociol. (2018) 77:1113–52. 10.1111/ajes.12230

[45] Yu L Lutz MW Wilson RS Burns DK Roses AD Saunders AM. APOE ε4-TOMM40 ‘523 haplotypes and the risk of Alzheimer's disease in older Caucasian and African Americans. PLoS ONE. (2017) 12:e0180356. 10.1371/journal.pone.0180356 28672022 PMC5495438

[46] Arvanitakis Z Bennett DA Wilson RS Barnes LL. Diabetes and cognitive systems in older black and white persons. Alzheimer Dis Assoc Disord. (2010) 24:37–42. 10.1097/WAD.0b013e3181a6bed5 19568148 PMC2837103

[47] Dore GA Waldstein SR Evans MK Zonderman AB. Associations between diabetes and cognitive function in socioeconomically diverse African Americans and whites. Psychosom Med. (2015) 77:643–52. 10.1097/PSY.0000000000000196 26163817 PMC4563816

[48] Hsu FC Sink KM Hugenschmidt CE Williamson JD Hughes TM Palmer ND. Cerebral structure and cognitive performance in African Americans and European Americans with type 2 diabetes. J Gerontol Ser A. (2018) 73:407–14. 10.1093/gerona/glx255 29309525 PMC5861881

[49] Kirchhoff BA Jundt DK Doty T Hershey T. A longitudinal investigation of cognitive function in children and adolescents with type 1 diabetes mellitus. Pediatr Diabetes. (2017) 18:443–9. 10.1111/pedi.12414 27444539 PMC5912686

[50] Lin A Northam EA Rankins D Werther GA Cameron FJ. Neuropsychological profiles of young people with type 1 diabetes 12 yr after disease onset. Pediatr Diabetes. (2010) 11:235–43. 10.1111/j.1399-5448.2009.00588.x 20070555

[51] Holder MK Blaustein JD. Puberty and adolescence as a time of vulnerability to stressors that alter neurobehavioral processes. Front Neuroendocrinol. (2014) 35:89–110. 10.1016/j.yfrne.2013.10.004 24184692 PMC3946873

