# Supplementary material for: Race and sex differences in the association between lifespan glycemic status and midlife cognitive function: the Bogalusa heart study
Source: Front Public Health. 2023 Nov 15;11:1200415. doi: 10.3389/fpubh.2023.1200415 (PMC10684774; doi:10.3389/fpubh.2023.1200415)
Supplement: Supplementary file 1 [file Table_1.DOCX]

| **Measure** | **Overall** | **Excluded Sample** |
| --- | --- | --- |
| N (Total) | 1292 | 6 |
| Sex (% Women) | 59% | 33% |
| Race (% African American) | 34% | 67% |
| Education | 13.32 +/- 2.48 | 13.33 ± 2.31 (n = 3) |
| **Childhood/Adolescence Epoch (≤20 years)** |  |  |
| Glucose (mg/dL) | 84.57 +/- 7.01  n=1229 | 82.97 +/- 10.41  n = 6 |
| Insulin (mg/dL) | 13.31 +/- 9.28  n=938 | 21.67 +/= 18.94  n=4 |
| HOMA-IR | 2.79 +/- 2.18  n=938 | 4.17 +/- 3.07  n=4 |
| **Early Adulthood Epoch (21-40 years)** |  |  |
| Glucose (mg/dL) | 85.87 +/- 18.06  n=1108 | 87.62 +/- 8.73  n=6 |
| Insulin (mg/dL) | 12.44 +/-9.7  n=1071 | 13.56 +/- 4.47  n=6 |
| HOMA-IR | 2.77 +/- 3.07  n=1057 | 2.91 +/- 1.12  n=6 |
| **Midlife (>40 years)** |  |  |
| Glucose (mg/dL) | 103.37 +/- 34.89  n=1173 | 107.17 +/- 15.05  n=4 |
| Insulin (mg/dL) | 12.93 +/- 9.99  n=472 | 19.45 +/- 7.57  n=2 |
| HOMA-IR | 3.21 +/- 3  n=470 | 5.51 +/- 2.86  n=2 |
